# Feasibility and initial experience of left radial approach for diagnostic neuroangiography

**DOI:** 10.1038/s41598-020-80064-z

**Published:** 2021-01-13

**Authors:** Nohra Chalouhi, Ahmad Sweid, Fadi Al Saiegh, Kalyan C. Sajja, Richard F. Schmidt, Michael B. Avery, Nikolaos Mouchtouris, Omaditya Khanna, Joshua H. Weinberg, Victor Romo, Stavropoula Tjoumakaris, Michael Reid Gooch, Nabeel Herial, Robert H. Rosenwasser, Pascal Jabbour

**Affiliations:** 1 Department of Neurosurgery, Chief Division of Neurovascular Surgery and Endovascular Neurosurgery, Thomas Jefferson University and Jefferson Hospital for Neuroscience, 901 Walnut street 3rd Floor, Philadelphia, PA 19107 USA; 2 Department of Anesthesia, Thomas Jefferson University and Jefferson Hospital for Neuroscience, Philadelphia, PA USA

**Keywords:** Neuroscience, Diseases, Neurology

## Abstract

Neuroangiography has seen a recent shift from transfemoral to transradial access. In transradial neuroangiography, the right dominant hand is the main access used. However, the left side may be used specifically for left posterior circulation pathologies and when right access cannot be used. This study describes our initial experience with left radial access for diagnostic neuroangiography and assesses the feasibility and safety of this technique. We performed a retrospective review of a prospective database of consecutive patients between April 2018 and January 2020, and identified 20 patients whom a left radial access was used for neurovascular procedures. Left transradial neuroangiography was successful in all 20 patients and provided the sought diagnostic information; no patient required conversion to right radial or femoral access. Pathology consisted of anterior circulation aneurysms in 17 patients (85%), brain tumor in 1 patient (5%), and intracranial atherosclerosis disease involving the middle cerebral artery in 2 patients (10%). The left radial artery was accessed at the anatomic snuffbox in 18 patients (90%) and the wrist in 2 patients (10%). A single vessel was accessed in 7 (35%), two vessels in 8 (40%), three vessels in 4 (20%), and four vessels in 1 (5%). Catheterization was successful in 71% of the cases for the right internal carotid artery and in only 7.7% for the left internal carotid artery. There were no instances of radial artery spasm, radial artery occlusion, or procedural complications. Our initial experience found the left transradial access to be a potentially feasible approach for diagnostic neuroangiography even beyond the left vertebral artery. The approach is strongly favored by patients but has significant limitations compared with the right-sided approach.

## Introduction

Cerebral angiography has historically been performed through the transfemoral approach. Recently, an increasing number of centers have transitioned from transfemoral to transradial cerebral angiography^1–8^. This was primarily fueled by the data from the cardiology literature showing lower access-related complications, lower mortality rates, and better patient satisfaction rates with the transradial approach^3,9–11^.

For coronary procedures, the right radial artery is more often employed than the left radial artery due to greater ease for the operator^12^. Likewise, in cerebral transradial angiography, access has been performed almost exclusively from the right side except for isolated left vertebral artery pathology or right radial artery anatomic limitations^3,13^. However, there could be certain advantages to left radial access as suggested by the cardiac literature^12,14,15^.

This study describes our initial experience with a left radial approach for diagnostic neuroangiography and assesses the feasibility and safety of this technique.

## Methods

### Patient population

The study protocol was approved by the Thomas Jefferson University Institutional Review Board. Patient consent was waived due to the nature of the study. We performed a retrospective review of a prospectively maintained database of consecutive patients between April 2018 and January 2020 and identified 20 patients whom a left radial access was used for neurovascular procedures. Patient data, procedural specifics, and procedural outcomes were prospectively collected.

### Radial artery catheterization technique

**Figure 1:**
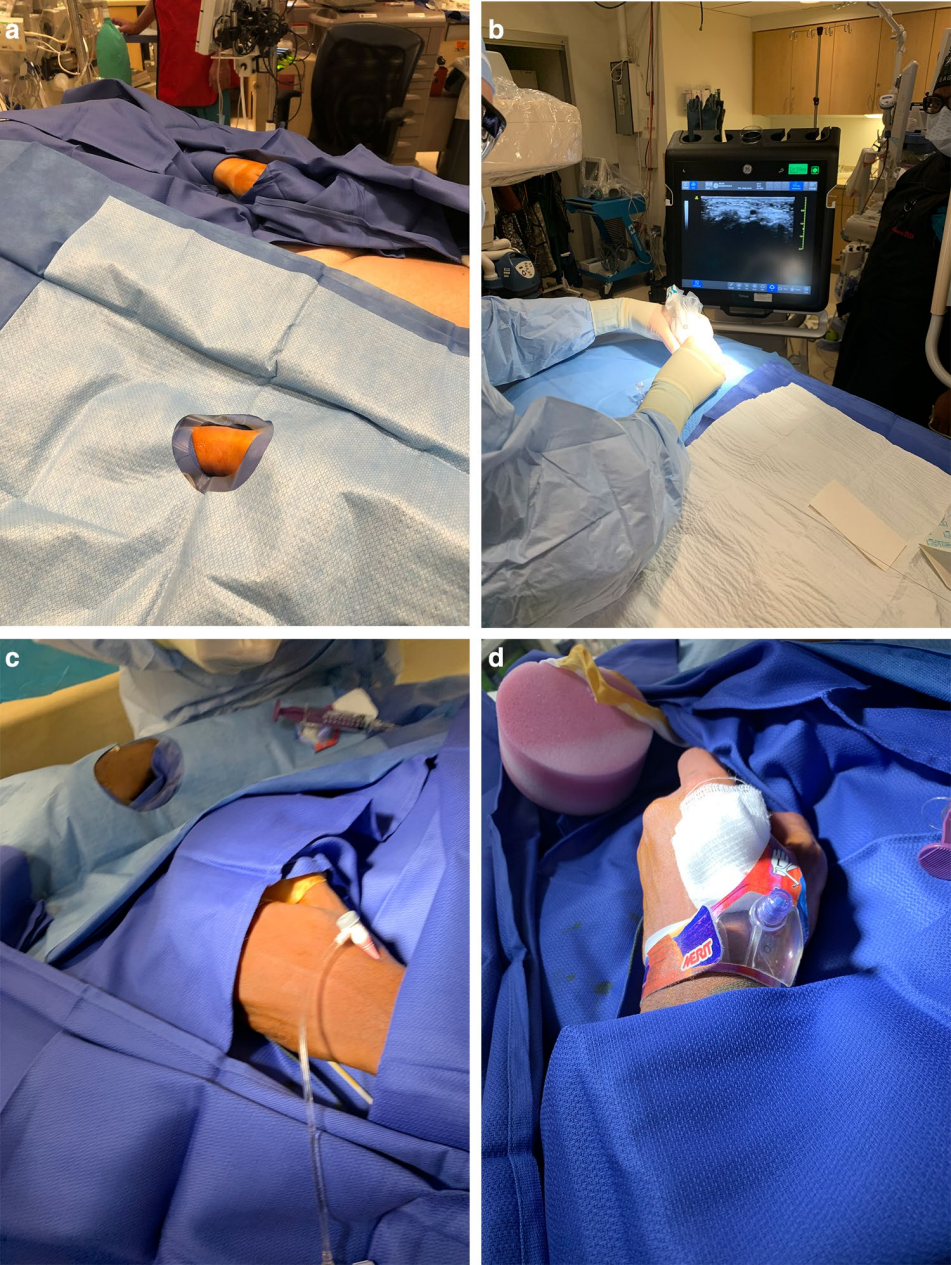
Pictures depicting our standard setup for left transradial neuroangiography. (**a**) Note the position of the left hand over the left groin, and the right wrist is prepped and draped as well. (**b**) Ultrasound guidance is utilized. (**c**) The sheath in inserted through the distal transradial artery “Snuff box approach.” (**d**) Closure is performed with the Prelude Sync radial compression device.

**Figure 2:**
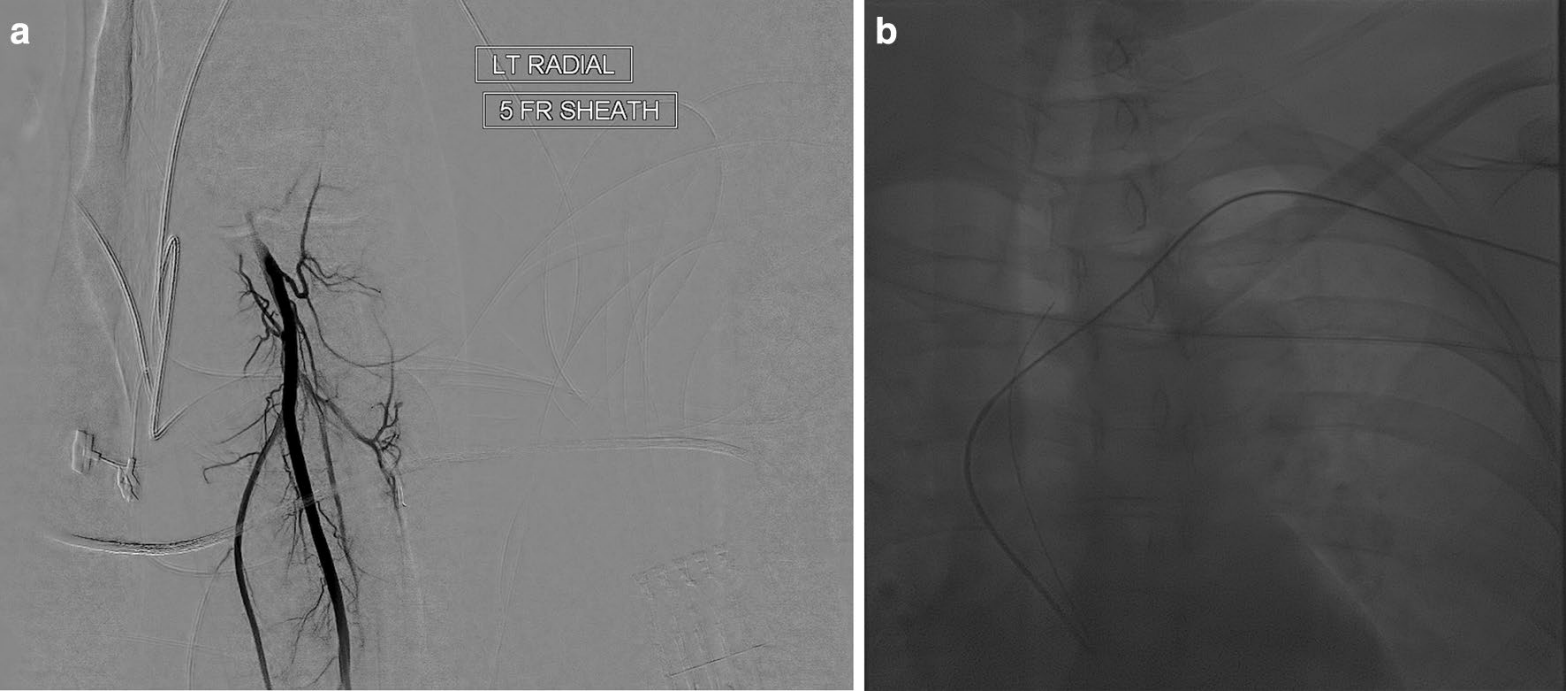
(**a**) A left radial artery angiogram is always performed. (**b**) AP view showing the process of reforming the catheter by bouncing the wire off the aortic valve. Note that the natural course of the wire from the left subclavian artery is the ascending aorta which facilitates reforming the catheter.

**Figure 3:**
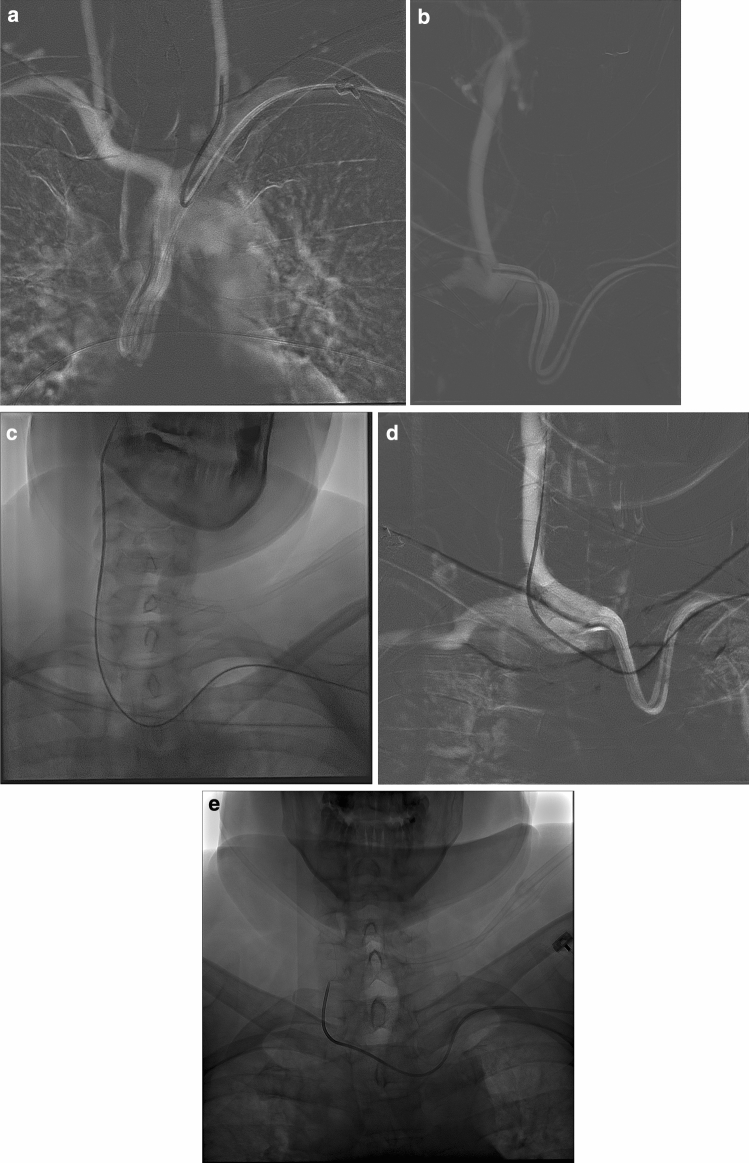
(**a**) AP views showing catheterization of the left common carotid artery, (**b**) the right common carotid artery, (**c**) the right internal carotid artery, (**d**,**e**) and the right vertebral artery from a left transradial approach.

All procedures are conducted using conscious sedation. The left wrist is positioned over the left groin to bring the access site closer to the operator standing on the right side. The left wrist is prepped and draped. The right wrist is positioned against the right hip of the patient in slight pronation and prepped and draped in the event that left radial access fails. Local lidocaine is administered in the left anatomic snuffbox, and the distal left radial artery is catheterized using ultrasound guidance via double wall puncture and Seldinger technique. If access at this site fails, the left radial artery is accessed at the wrist. Catheterization is achieved using a 5-French Prelude sheath. A mix of 2000 units of heparin, 5 mg of nicardipine, and 200 mcg of nitroglycerin is administered through the sheath. A radial run is then performed to evaluate the anatomy of the left radial artery. A 5-French Simmons 2 Penumbra catheter (Penumbra, Alameda, California, USA) is used to select the target vessels in its formed configuration in a similar fashion to right transradial angiography. After the procedure is complete, the sheath is removed and a radial artery compression device is applied (Figs. 1, 2 and 3).

### Ethical approval

All procedures performed in the studies involving human participants were in accordance with the ethical standards of the Institutional Review Board (IRB) or national research committee and with the 1964 Helsinki Declaration and its later amendments or comparable ethical standards.

### Informed consent

The study protocol was reviewed and approved by the Thomas Jefferson University Institutional Review Board. Following our institutional guidelines, all protected health information was removed and individual patient consents were not required for the analysis of this case series.

## Results

A total of 20 consecutive patients undergoing 20 procedures were included. Left transradial neuroangiography was successful in all 20 patients and provided the sought diagnostic information; no patient required conversion to right radial or femoral access.

Mean age was 56.7 ± 12.9 years and 75% (15/20) were female. The left radial artery was accessed at the anatomic snuffbox in 18 patients (90%) and the wrist in 2 patients (10%). Pathology consisted of anterior circulation aneurysms in 17 patients (85%), brain tumor in 1 patient (5%), and intracranial atherosclerosis disease involving the middle cerebral artery in 2 patients (10%). No patient had a vertebrobasilar pathology.

The catheter was reformed off the aortic valve in all 20 patients. The great vessels catheterized are listed in Table 1. A single vessel was accessed in 7 (35%), two vessels in 8 (40%), three vessels in 4 (20%), and four vessels in 1 (5%). Catheterization of the right internal carotid artery was successful in 71% (10/14). However, catheterization of the left internal carotid artery was successful in only 7.7% (1/13). There were no instances of radial artery spasm, radial artery occlusion, or procedural complications.

**Table 1:** Great vessels catheterized.

Access	Vessels accessed
Proximal radial	R ICA
Proximal radial	R ICA, L CCA
Distal radial	R CCA, L CCA, L VA
Distal radial	R ICA, R ECA, L CCA, L VA
Distal radial	R CCA, L CCA, L VA
Distal radial	R ICA, R VA
Distal radial	R CCA, L CCA, L VA
Distal radial	L CCA, L VA
Distal radial	R ICA
Distal radial	L CCA
Distal radial	R ICA, L ICA
Distal radial	L VA, R ICA, L CCA
Distal radial	L CCA
Distal radial	R ICA, L CCA
Distal radial	R ICA, L CCA
Distal radial	R ICA
Distal radial	R CCA
Distal radial	R CCA, L CCA
Distal radial	L CCA
Distal radial	R CCA, L CCA

*CCA* Common Carotid Artery, *ICA* Internal Carotid Artery, *VA* Vertebral Artery, *R* Right, *L* Left.

## Discussion

Cerebral angiography has traditionally been performed through the femoral artery. However, several large trials in interventional cardiology demonstrated a better safety profile of the transradial approach. The MATRIX trial showed in 8,404 patients that transradial angiography had a lower risk of mortality and major bleeding when compared to the transfemoral approach^16^. Feldman et al^17^ conducted a large-scale retrospective analysis of 2.8 million coronary interventions and found the risk of bleeding and vascular complications to be consistently lower using the transradial access compared to transfemoral access. Furthermore, the transradial approach seems to be preferred by patients as well. As many as 94% of patients who had cerebral angiography through both approaches favor transradial over transfemoral access due to a shorter recovery time, lower bleeding risk, and early post-procedural ambulation^3^. Taken together, these benefits have encouraged many neurointerventionalists to adopt the transradial approach for diagnostic angiography and neurointerventions including mechanical thrombectomy for stroke^18^.

Transradial neuroangiography is typically performed from the right side, but there is emerging evidence in interventional cardiology in favor of left-sided radial artery catheterization. Perhaps the most obvious advantage of the left-sided approach is the use of the non-dominant wrist in most patients, which allows the access site to heal with less restrictions in day-to-day activities. However, side-specific variations in vascular anatomy also make the left-sided radial approach more compelling. Various clinical trials have shown that the right subclavian artery has a higher incidence of tortuosity compared to the left subclavian artery^15^. In fact, Norgaz et al^14^ report an incidence of right-sided subclavian tortuosity that is almost 3-times as high compared to the left-sided subclavian artery. This can make navigating the catheter more challenging, thus, leading to an increased fluoroscopy time and higher radiation exposure as shown in the randomized controlled TALENT trial that included 1,467 patients undergoing coronary interventions^19^. A lower radiation exposure to the operator was also reported in another trial that compared left to right radial artery catheterization for coronary angiography^20^. Importantly, a study on diagnostic coronary angiography found a higher risk of cerebral embolization when the procedure was performed from the right side^21^. Further research is needed to determine if this finding applies to neuroangiography as well.

As discussed above, a key advantage of left radial neuroangiography is the possibility of accessing the non-dominant hand in right-handed patients. We prefer to use the snuffbox over the traditional approach as it allows more ergonomic left hand positioning over the groin and obviates the need for hand supination and taping. An additional advantage that we encountered with left radial neuroangiography is the ease with which the Simmons 2 catheter can be formed as the wire will almost always travel to the ascending aorta allowing the catheter to be readily formed off the aortic valve. This has the potential to save fluoroscopy time as forming the Simmons catheter from the right radial artery can prove challenging at times especially when the wire keeps directing down into the descending aorta. It is also more efficient to catheterize the great vessels from the ascending as opposed to the descending aorta. Additionally, we have found that, when performing the radial artery run, left radial access obviates the need to rotate the fluoroscopy table as is necessary on the right side since the left wrist is positioned within the anterior–posterior plane over the left groin. Another potential advantage of the left transradial approach is its relative ease in accessing the ipsilateral vertebral artery which is dominant in most individuals^22^. In such cases, catheter shaping is not required as in the right radial approach. Given the position of the left hand in the center of the operative field, spillage of blood onto the floor is less likely to happen with left radial access compared with radial access. Although arteria lusoria (the prevalence of which is about 1%) is considered a contraindication to right transradial neuroangiography^6^, a left transradial approach should allow easy catheterization of the great vessels in these individuals.

Our results demonstrate the safety and technical feasibility—albeit with limitations—of the left transradial approach for diagnostic neuroangiography beyond the left vertebral artery. We found that the left radial approach allows efficient catheterization of the right and left common carotid arteries after reforming the Simmons catheter. However, the major shortcomings we found in our series is the difficulty in catheterizing the right internal carotid artery (successful in 71%) and especially the left internal carotid artery (successful in only 1 patient). The latter limitation is due to the acute angle between the left subclavian artery and left common carotid artery. For this reason, we do not recommend using left radial access when selection of the left internal or external carotid arteries is needed. This shortcoming may be mitigated in the future by catheters and wires specifically designed for transradial access. Another limitation we found with left transradial access is the distance between the access site and the operator especially in obese patients. Hand positioning over the left groin and use of a long catheter such as the Simmons 2 Penumbra catheter (available in 125 cm and 130 cm) help counter this issue. Because of these limitations, the right side should remain the first line approach for transradial angiography.

In a recently published study, Barros et al^13^ described their experience with left transradial neuroangiography in 19 patients who underwent 25 procedures. In 16 of these patients, left radial access was chosen to address left vertebral pathology and, in contrast with our study, only 5 patients underwent catheterization of the carotid system. The authors did not report the success rate for selection of the great vessels. They also utilized the left radial artery only when right-sided access was not feasible. Specifically, the indications for left radial access were left vertebral pathology/dominance, right subclavian stenosis, right radial artery pathology, or traumatic amputation of the right upper extremity. The authors concluded that left transradial access in cerebral angiography is safe and feasible but may be reserved for patients with anatomic limitations of the right radial artery or left vertebral pathology/dominance.

The limitation of this study is that it is a retrospective, pilot study composed of a small study group. In addition, the studied cohort lacked a control group to compare outcomes. Further studies composed of a larger cohort and a control group are warranted to validate our results.

## Conclusion

Overall, our initial experience found the left transradial access to be a potentially feasible approach for diagnostic neuroangiography even beyond the left vertebral artery. The approach is strongly favored by patients but has significant disadvantages as compared with the right-sided approach, the most important of which is the inability to catheterize the left internal carotid artery. Transradial diagnostic neuroangiography and neurointerventions should continue to be performed through the right radial artery preferentially but future efforts should focus on developing better wires and catheters to improve the efficacy of left radial artery neuroangiography.

## Data Availability

The relevant anonymised patient level data are available on reasonable request from the authors.
